# Evaluation of the analytical and diagnostic performance of a digital droplet polymerase chain reaction (ddPCR) assay to detect *Trypanosoma cruzi* DNA in blood samples

**DOI:** 10.1371/journal.pntd.0007063

**Published:** 2018-12-26

**Authors:** Juan David Ramírez, Giovanny Herrera, Carolina Hernández, Lissa Cruz-Saavedra, Marina Muñoz, Carolina Flórez, Robert Butcher

**Affiliations:** 1 Grupo de Investigaciones Microbiológicas-UR (GIMUR), Programa de Biología, Facultad de Ciencias Naturales y Matemáticas, Universidad del Rosario, Bogotá, Colombia; 2 Grupo de Parasitología, Instituto Nacional de Salud, Bogotá, Colombia; 3 Clinical Research Department, London School of Hygiene & Tropical Medicine, London, United Kingdom; University of Georgia, UNITED STATES

## Abstract

**Background:**

The recent development of novel Polymerase Chain Reaction (PCR) technologies that confer theoretical advantages over quantitative PCR has considerable potential in the diagnosis of low load infections, such as *Trypanosoma cruzi* in the chronic phase of Chagas disease. We evaluated the utility of the digital droplet (dd)PCR platform in the detection of *T*. *cruzi* infection.

**Methodology/Principal findings:**

We imported a validated qPCR assay targeting the *T*. *cruzi* satellite tandem repeat (TcSTR) region to the ddPCR platform. Following optimization, we tested and repeated a standard curve of TcI epimastigotes to characterise the analytical performance of the assay on the ddPCR platform. We compared this to published qPCR performance data, and the performance of the qPCR assay in our own testing. We subsequently tested a panel of 192 previously characterized DNA specimens, extracted from the blood of individuals with and without *T*. *cruzi* infection. The assay performed well on the ddPCR platform, showing a limit of detection of 5 copies/μL or 1 parasite/mL. This was higher than the published limit of detection for qPCR, which was 0.46 parasites/mL. The ddPCR platform was not significantly more accurate than qPCR at any concentration tested. However, the clinical sensitivity and specificity of the assay were both 100% with perfect agreement between qPCR and ddPCR positive and negative result calling in clinical specimens. An average of 9,286 copies of TcSTR were detected per parasite.

**Conclusions/Significance:**

The use of the ddPCR platform to run this assay was comparable, but not superior in terms of performance, to the qPCR platform.

## Author summary

Chagas disease is a complex pathology caused by the parasite *Trypanosoma cruzi*. This disease affects more than 8 million people in Latin America and is considered a serious public health problem. The diagnosis of the disease is challenging due to the natural course of infection and therefore molecular tools such as qPCR have been deployed to detect parasite DNA in infected individuals. However, qPCR is not suitable for diagnosis in the chronic phase of the disease and new diagnostic tools are needed. Digital droplet (dd)PCR is a next-generation PCR technology that enables the absolute quantitation of nucleic acids. This technology has been successfully applied as a diagnostic tool in bacteria and viruses, but few reports exist for parasites. Herein, we imported a previously validated qPCR assay to the ddPCR platform. Our results show complete agreement in terms of diagnostic operative features but a higher limit of detection. Overall, ddPCR did not offer significant diagnostic benefits compared with qPCR.

## Introduction

Chagas disease, caused by *Trypanosoma cruzi*, is a complex chronic pathology that affects around 8 million people worldwide and represents a serious public health problem [1]. *T*. *cruzi* parasites exhibit tremendous within-species genetic diversity and have been subdivided by international consensus into at least six discrete typing units (DTUs) from TcI to TcVI [2, 3]. The acute phase of the disease is usually characterized by mild fever, but in a small proportion of cases, it can be accompanied by myocarditis and other lethal complications [4]. Most patients continue into the chronic phase, which can be asymptomatic, but about 30% of the infected patients will develop heart or digestive complications after several years [5].

The diagnosis of Chagas disease is challenging. In the acute phase of infection and disease, the parasitic load is sufficiently high that direct methods of parasitological observation such as a blood smear and hematocrit are recommended to diagnose the disease [6–8]. By contrast, the chronic phase is characterized by a low and intermittent parasitic load that is not likely to be detected using direct parasitological methods. Therefore, serological assays such as indirect immunofluorescence assays, indirect hemagglutination test and enzyme linked immunosorbent assays (ELISA) are recommended to diagnose the disease [9–12]. Molecular methods have been developed and currently, there are several real-time quantitative PCR assays that are commonly used internationally to detect and quantify *T*. *cruzi* DNA in blood samples of acute and chronic Chagas disease patients [13–15]. The evaluation of a number of different genetic sequences suggested a tandem repeat satellite DNA (TcSTR) sequence of 188 bp that is continuously repeated across the genome of *T*. *cruzi* to be an appropriate qPCR target for optimal sensitivity and specificity [14].

The operating characteristics of molecular tests for detecting *T*. *cruzi* DNA have varied according to clinical phase and technical specifications. The sensitivity for identifying chronic infection of end-point PCR has ranged between 22% and 75% [9, 16] and in both cases specificity has estimated at 100%. By contrast, for quantitative PCR (qPCR), sensitivity has ranged between 60% and 80% for the chronic phase and between 88% and 100% for the acute phase of infection [6, 14]. Recently, a loop-mediated isothermal amplification assay (LAMP) was reported that was able to detect *T*. *cruzi* DNA in peripheral blood samples collected from well-characterised seropositive patients, including acute, congenital, chronic and reactivated Chagas disease [17, 18]. The sensitivity of qPCR in the acute phase makes it ideal for the detection of such infections; however, the low levels or absence of circulating parasites in chronic cases means that the sensitivity of this assay is currently too low to allow for proactive screening for patients with chronic infection. The development of an assay with better performance at low concentrations may therefore enable the improved detection of infection in chronic Chagas disease cases, which may support case findings and monitoring.

Digital droplet PCR (ddPCR) is a next-generation PCR technology that enables the absolute quantitation of nucleic acids by separating a reaction into ~20,000 compartments (droplets) and using the proportion of fluorescent droplets to indicate target occupancy and positivity. By isolating the PCR reaction into compartments, the relative concentration of targets, primers and probes is high, and of inhibitors is low, thereby theoretically allowing improved precision at lower concentrations and an improved analytical limit of detection compared with qPCR. Additionally, ddPCR measures the response to the number of copies present in a linear manner thereby allowing smaller differences in load to be accurately quantified, compared with qPCR that measures logarithmic increases in the target number. The theoretical benefits of this technology have encouraged its evaluation in the detection of pathogens [19]. While ddPCR has been evaluated for the detection of bacteria and viruses (for example, *Chlamydia trachomatis*, *Salmonella*, *Escherichia coli*, *Campylobacter jejuni*, cytomegalovirus and HIV) [20–24], few investigations have been conducted into the role of ddPCR in the detection of protozoan infections. ddPCR assays have been initiated for *Schistosoma*, *Cryptosporidium* and *Plasmodium* [25–27]. Therefore, the aim of this work was to import a validated international consensus qPCR assay targeting the TcSTR [14] to the ddPCR platform and compare its analytical and diagnostic performance with the qPCR iteration to determine whether the technology may be useful.

## Methods

### Ethical statement

The study protocol was approved by the Technical Research Committee and Ethics Research Board at the National Health Institute in Bogotá, Colombia, protocol CTIN-023-17 in the framework of the project “Fortalecimiento y vigilancia de la capacidad diagnóstica de enfermedades emergentes y reemergentes en Colombia”. Participation (adults only) was voluntary and patients were asked for informed written consent. The diagnostic evaluation protocol was additionally approved by the London School of Hygiene & Tropical Medicine Observational Ethics Committee (protocol number 15515).

### Preparation of standard curves for *T*. *cruzi*

*T*. *cruzi* parasites of TcI (MHOM/CO/04/MG) and TcII (MHOM/BR/53/Y) from different life-cycle stages were grown (epimastigotes and metacyclic trypomastigotes) in culture as reported elsewhere [28]. Cultures were aliquoted in the exponential growth stage. A Neubauer chamber was used to assess the concentration of parasites per mL. Then, 300 μL of the counted parasites were subjected to DNA extraction using the High Pure PCR Template Roche kit as reported elsewhere [29], and the DNA was eluted in 100 μL of H_2_O or TE buffer.

### qPCR for *T*. *cruzi*

The satellite tandem repeat of *T*. *cruzi* (TcSTR) was selected due to its high copy number in the genome and its routine usage as a diagnostic target for *T*. *cruzi* infection. The TcSTR region can be repeated up to 10^5^ times in the genome and can account for as much as 10% of the genetic material, although this varies between DTUs. The qPCR described by Ramirez *et al*. was considered an appropriate comparator for this study due to the availability of detailed evaluation data of analytical and diagnostic performance [14]. For this study, qPCR was conducted using published methodology. The standard curve for TcI epimastigote parasite material was determined with the qPCR assay on a single plate using five technical replicates.

### ddPCR assay preparation

The TcSTR sequences from Ramirez *et al*. [14] were used in this study. An endogenous control assay (*Homo sapiens* ribonuclease P/MRP subunit p30 [HsRPP30] as described by Luo *et al*. 2005 [30]) was also added to the test to enable users to check that specimen collection and sample processing had been completed successfully (Table 1). Both TcSTR and HsRPP30 assays were run in duplicate in a single well. The ddPCR reaction mixtures (20 μL volume) contained final concentrations of 1× ddPCR Supermix (Bio-Rad, Hemel Hempstead, UK), 0.9 μM for each primer and 0.2 μM for probes, and 5 μL of purified sample DNA (Table 1). Droplet generation and droplet reading for ddPCR were carried out using the Bio-Rad QX100 workflow according to the manufacturer's instructions. The first thermal cycling profile tested was matched to the published qPCR protocol (95°C for 10 minutes followed by 45 cycles of 95°C for 15 seconds and 60°C for 30 seconds). However, under these cycling conditions for ddPCR, the primer–probe set yielded poor separation between positive and negative droplet populations. Based on the results of a systematic assessment of parameters to improve ddPCR performance [31], the dissociation and annealing/extension steps in the thermocycling conditions were extended to 30 seconds and 2 minutes, respectively, and the number of cycles was increased to 50.

**Table 1:** Sequences of the primers and probes used in the ddPCR assay.

Primer/probe	Sequence 5ʹ-3ʹ	Reference
cruzi1	ASTCGGCTGATCGTTTTCGA	[14]
cruzi2	AATTCCTCCAAGCAGCGGATA	[14]
cruzi3	FAM-CACACACTGGACACCAA-MFQB	[14]
HsRPP30Fw	AGATTTGGACCTGCGAGCG	[30]
HsRPP30Rv	GAGCGGCTGTCTCCACAAGT	[30]
HsRPP30 Probe	Hex-TTCTGACCTGAAGGCTCTGCGCG-Qsy-7	[30]

### ddPCR data processing

Raw ddPCR data were collected using Quantalife software (Bio-Rad) and were then exported for analysis using R scripts as reported elsewhere [20]. Thresholds between positive and negative droplet populations were set manually using per-plate positive and no-template controls as a guide; for the TcSTR assay, droplets with an amplitude above 3,000 were considered positive. The Poisson calculation was used to estimate the number of copies/μL of the reaction and confidence intervals [32]. For classification purposes, we followed the method described by Roberts *et al*. to optimize sensitivity and specificity [20]. We used the estimated mean concentration of the target and its standard deviation to define the cumulative distribution function (c.d.f.) at x = 0. This value describes the probability that the true concentration is less than or equal to zero copies/μL. The classifier ζ was defined as 1 ˗ c.d.f. and describes the probability that the true concentration is greater than zero copies/μL. We excluded samples from further analysis if the ζ value for the RPP30 assay was below 0.95. A specimen was considered positive for ddPCR when the lower 95% confidence interval of the load estimate was above zero, which is considered to optimize specificity as described by Roberts *et al*. 2013 [20]. Samples with over 99% droplet occupancy were considered too concentrated for accurate quantitation as they potentially contravened assumptions of the Poisson distribution. While binary diagnostic classification is possible, these specimens were retested at a 1 in 100 dilution for quantitation purposes.

### ddPCR assay analytical performance

A standard curve of TcI epimastigote-stage *T*. *cruzi* parasites diluted 10-fold from 10^4^–10^−3^ parasites/mL was repeat-tested to assess the reproducibility of the ddPCR test. Each dilution in the series was tested in five technical replicate wells on a single plate to determine within-assay variance and then in a single well on five different plates to assess between-plate variability. The analytical limit of detection was determined as the concentration of the lowest step of this series at which all 10 replicates tested positive. Due to the known variability in copy number of the TcSTR region between DTUs [33], we also tested dilution series of TcII parasites to ensure the assay would not be affected by the differing population genetics of *T*. *cruzi*. A dilution series of the TcI trypomastigote-stage was also tested to ensure that the blood stage of the parasite was detectable. To estimate the correlation in quantitation between qPCR and ddPCR, the concentration estimates from both platforms were tested using the Pearson correlation coefficient. The number of TcSTR copies per parasite was determined using ddPCR. The number of parasites per extraction was 0.3 parasites per mL, as 300 μL of parasite culture was used in the extraction protocol and downstream analysis. The number of copies per μL in the ddPCR reaction was also multiplied by four to account for the dilution of DNA in the reaction and was used to quantify the number of TcSTR copies in each dilution point of the standard curve material.

### ddPCR diagnostic performance

A total of 192 samples were included in the analysis. Blood samples were collected and stored in 6 M guanidine hydrochloride and 0.2 M EDTA buffer, pH 8, as described elsewhere [9]. Multiplex qPCR for the detection of satellite DNA of *T*. *cruzi* was performed as reported elsewhere [34]. The parasitic load was measured by qPCR as parasites per mL according to Moreira *et al*. 2013 [29]. A total of 106 positive samples that were previously characterized by qPCR, indirect immunofluorescence assays, an indirect hemagglutination test and ELISA as positive and 86 negative samples were also characterized by the same techniques.

Our samples included those from patients in the acute phase of infection (n = 11; 10.4%). A suspected acute case was defined as an individual with >7 days of fever accompanied or not by hepatomegaly or splenomegaly. A patient was considered to have acute Chagas disease if in addition to testing positive in parasitological tests (strout, micro-strout, blood thick smear or hemoculture), positive results were obtained for two serological tests over the course of the following weeks. The average patient age was 49 years (from 5 to 61 years old).

Our samples also included those from patients in the chronic phase of infection (n = 95; 89.6%). These individuals did not fulfil the criteria for acute phase infection but had clinical or epidemiological suspicion of Chagas disease. The average age of these patients was 49 years (from 26 to 88 years old). These patients were confirmed as positive for *T*. *cruzi* infection when they tested positive to two serological tests (IFA, ELISA and/or HAI). They were then classified as chronic undetermined (when no evidence of signs or symptoms of heart complications were evinced) or chronic determined in all other cases. Among the set of 95 chronic patients, a total of 30 patients were symptomatic (determined) and the other 65 patients were asymptomatic (undetermined).

Descriptive analyzes were performed to report the frequencies of infection detected by qPCR and ddPCR, considering the qualitative result (positive/negative) for each test with respect to the total number of samples evaluated (n = 192). The agreement between the results of the tests was evaluated through the calculation of the coefficient kappa. Sensitivity, specificity and positive and negative predictive values were determined, as indicators of the operative characteristics of the ddPCR, taking as a reference test the qPCR, previously identified as being efficient for the detection of infection [14]. A 95% confidence interval (95% CI) using the analytical method (due to the dichotomous nature of the variables analyzed), was calculated for the main events of interest. A value of P<0.05 was considered significant. The statistical analyzes were performed using the software STATA 12.0.

Finally, to test the association between parasitic loads using both platforms (qPCR vs. ddPCR), a graphic of Bland–Altman was generated using Microsoft Office Excel 2016 software. For this, the differences between the loads calculated by ddPCR and qPCR were determined, and these two values were averaged for each of the samples. Bias was then calculated by means of the average of the differences for all samples, as well as their respective standard deviations. The upper and lower limits of the deviation were calculated using the bias +/˗ 1.96 multiplied by the standard deviation. For the statistical tests, normality tests (Shapiro–Wilk) were initially carried out to determine the distribution of the data. Based on their distribution, the correlations between age and parasitic loads calculated by each of the evaluated methods were determined. The differences in the medians of the parasitic loads calculated by each of the methods, both general and according to the clinical phase and state of the disease were determined using a Mann–Whitney U test. All statistical analyzes were performed using the Stata 14 software (StataCorp, 2015. Stata Statistical Software: Release 14. College Station, TX: StataCorp LP).

## Results

### ddPCR assay optimisation

**Fig 1:**
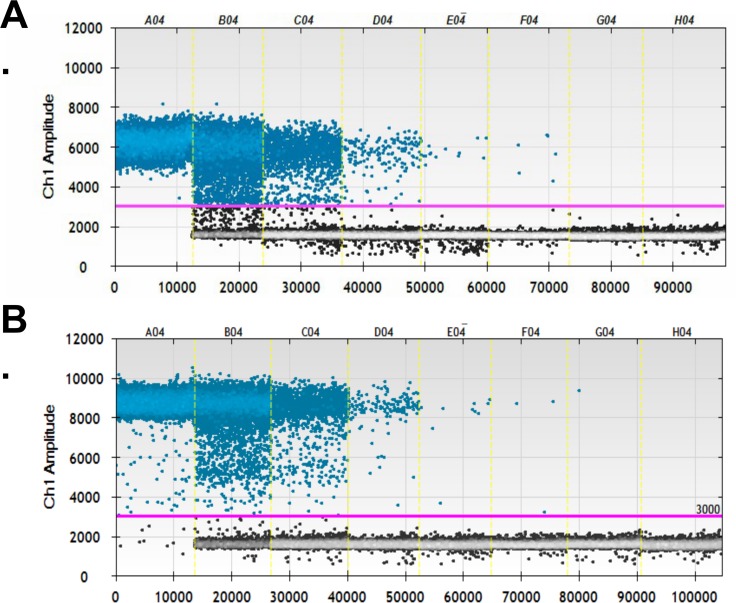
**Droplet amplitude in the *T. cruzi* satellite tandem repeat assay on the ddPCR platform for a range of concentrations (wells A–G) and the no-template control (well H).** (A) Assay performed under previously published qPCR thermocycling conditions (40 cycles of 95°C for 15 seconds and 60°C for 30 seconds). (B) Assay performed under optimized conditions (50 cycles of 95°C for 30 seconds and 60°C for 120 seconds).

The thermal cycling conditions for the TcSTR ddPCR assay were optimized for this study. Fig 1 shows (A) the native assay conditions and (B) the optimised assay conditions across a standard curve (wells A–G) and one no-template control well (well H). In the diagnostic target (TcSTR) channel (FAM fluorophore), there was a small increase in the mean negative population amplitude under optimized cycling conditions compared with the native cycling conditions (1599 versus 1554, p = 0.01). There was a large increase in mean amplitude of the positive droplets under the optimized cycling conditions compared with the native ones (8255 versus 5027, p = 0.002). The ratio of positive-to-negative droplet amplitude also increased from 3.2 to 5.2 (p = 0.002). No-template control wells were negative for both channels under optimized and native thermocycling conditions. There was no difference in the endogenous control channel HsRPP30 (HEX fluorophore) negative droplet amplitude between optimised and native conditions (1161 versus 1146, p = 0.10).

### ddPCR analytical performance

During repeat testing of the *T*. *cruzi* standard curve, the top standard (10^4^ parasites/mL) resulted in over 99% of the droplets being saturated. The upper limit of the dynamic range for accurate quantitation of this assay was 10^3^ parasites/mL. Of 10 repeated readings of the 1 parasite/mL standard, all 10 were detected. Of 10 repeat readings of the 0.1 parasites/mL standard (equivalent to approximately 0.5 TcSTR copies/μL or 2.5 TcSTR copies/test), only seven were detected. The lower dilutions were also not detected in all repeats; therefore, the limit of detection is 1 parasite/mL, measured as 5 TcSTR copies/μL or 20 TcSTR copies/test. The TcI epimastigotes tested had a mean of 9,286 (range: 8,768–10,113) TcSTR copies per parasite.

**Fig 2:**
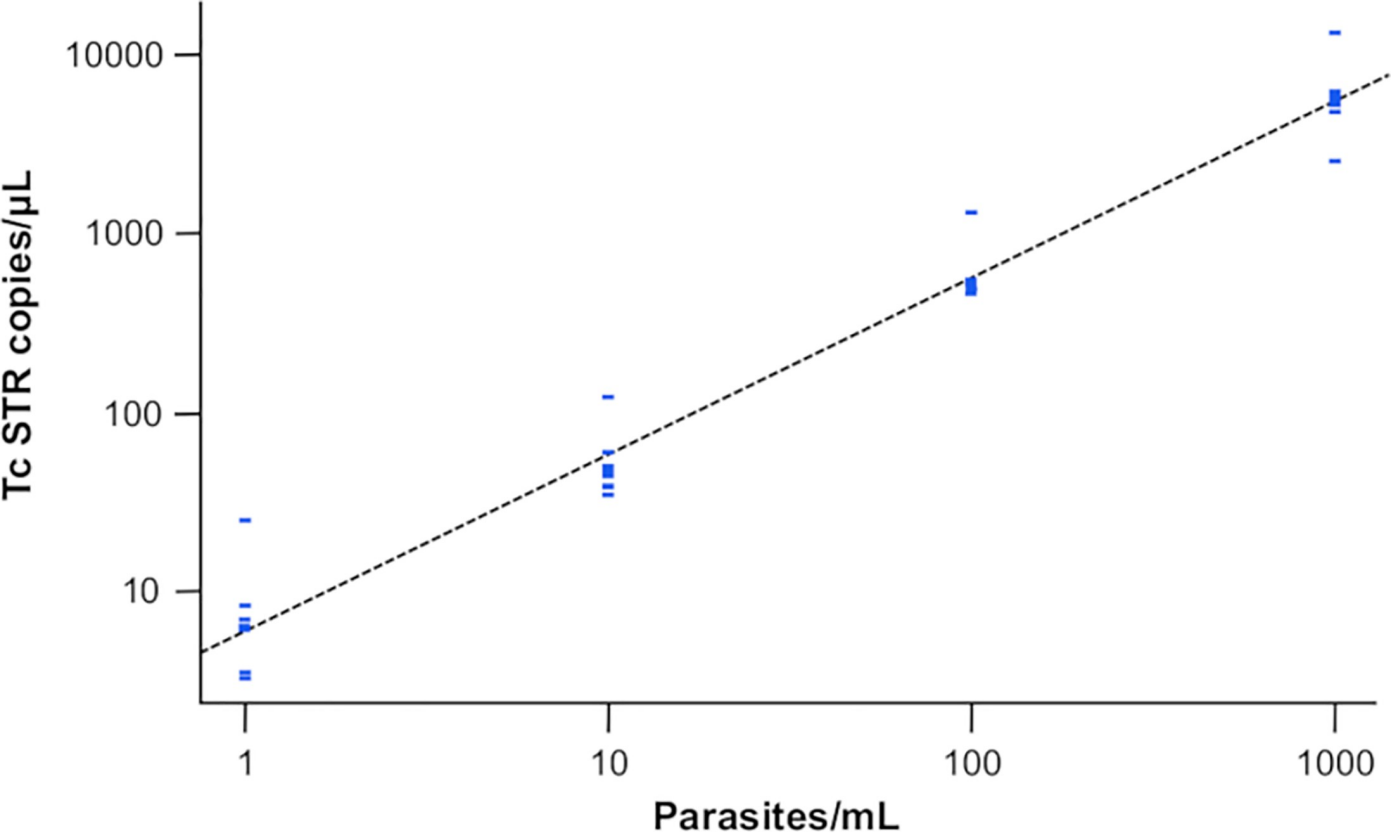
Serial 10-fold dilution of TcI epimastigote-stage *T*. *cruzi* repeat tested with the *T*. *cruzi* satellite tandem repeat assay on the ddPCR platform.

**Fig 3:**
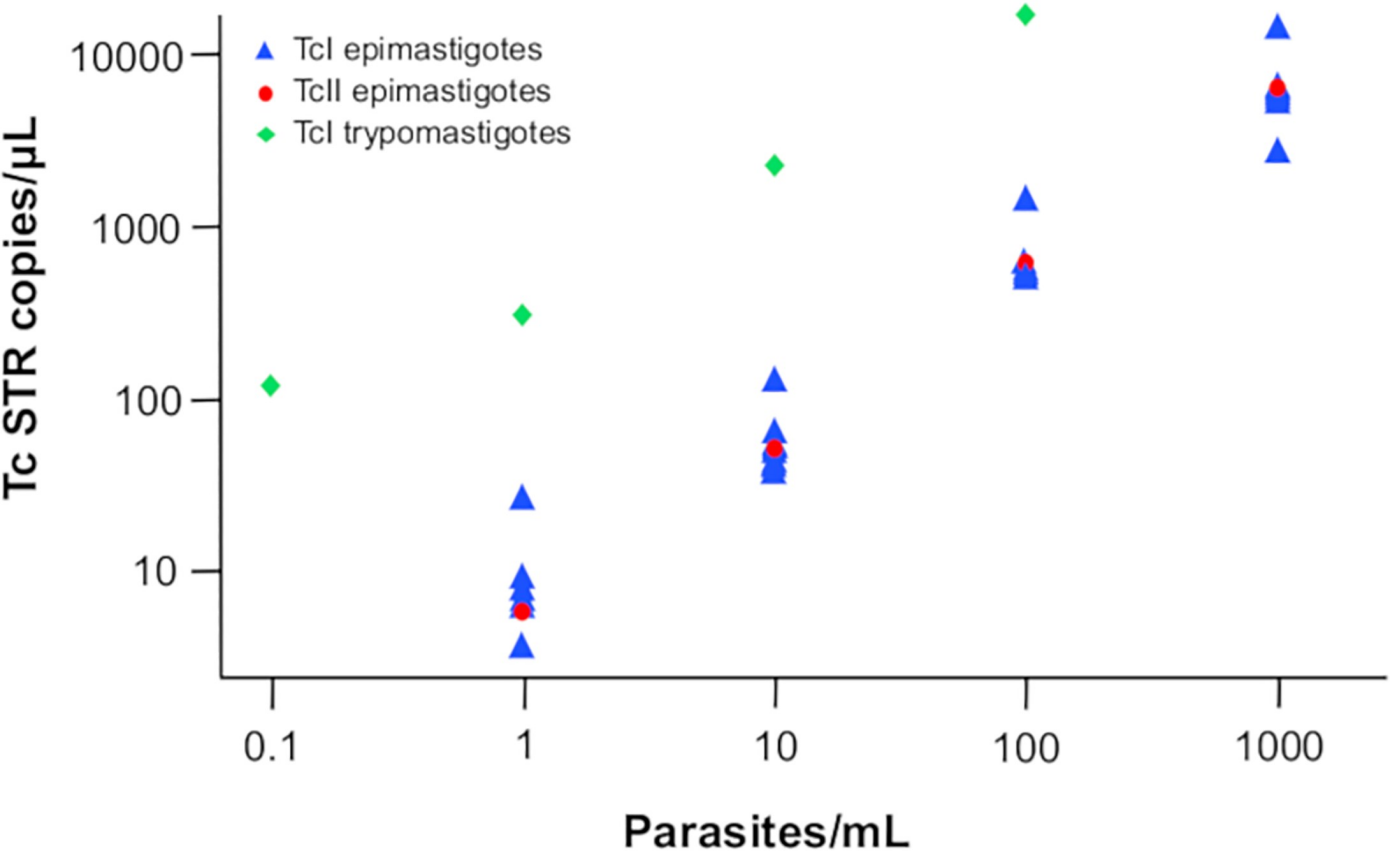
Serial 10-fold dilution of TcII epimastigote-stage and TcI trypomastigote (blood) stage *T*. *cruzi* repeat tested with the *T*. *cruzi* satellite tandem repeat assay on the ddPCR platform.

The Pearson correlation coefficient between TcSTR copies/μL and parasites/mL in the dynamic range of the dilution series was 0.987. The efficiency of the assay was 98% (Fig 2). The correlation between the qPCR cycle threshold and the ddPCR load estimate was excellent in the standard curve. Upon testing of different developmental stages and different lineages, both TcII and TcI trypomastigote curves also performed equally well as the TcI epimastigote curve (Fig 3).

**Fig 4:**
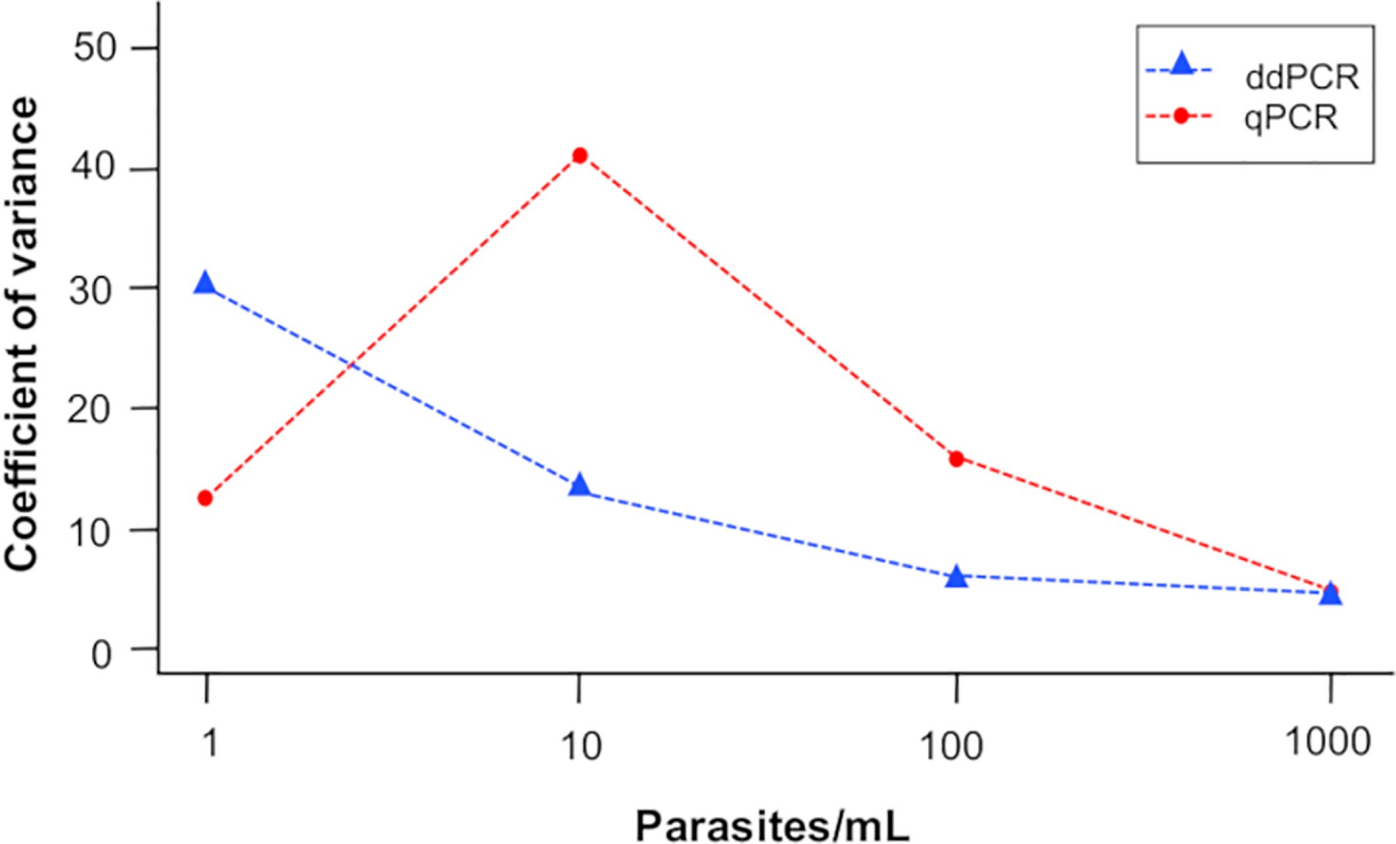
Within-plate variance of the *T*. *cruzi* satellite tandem repeat assay performed on the qPCR and ddPCR platforms across a range of parasite loads.

The ddPCR between-plate coefficient of variation (CV) across the whole series was 101%, and the CV at 1 parasite/mL was much higher than at 1000 parasites/mL (200% compared with 53%). The within-plate CV was 13%, and the CV at 1 parasite/mL was much higher than at 1000 parasites/mL (30% compared with 4%). When comparing the within-plate CV between qPCR and ddPCR, the CV of ddPCR across the dilution series did not differ between the platforms (qPCR CV = 18.6% versus ddPCR CV = 13.0%). Also, the ddPCR CV was not significantly lower than the CV of qPCR at the lower end of the dilution series (Fig 4).

### ddPCR diagnostic performance

The frequency of positives was the same for the two tests being evaluated, at 55.2% [n = 106; 95% CI = 47.9–62.4]. The concordance between the results was absolute (100%) and was supported by a perfect kappa coefficient (1.0000). Analysis of the operational characteristics of the ddPCR test revealed great performance (100% in all cases) in detecting *T*. *cruzi* DNA (Table 2), when compared with the results of the qPCR test included as a reference. For all clinical phases and totals there was absolute agreement between the tests. All positive samples were correctly classified, as were the negative samples. Therefore, all values accurately classified the samples by clinical stage (100%).

**Table 2:** Operative characteristics of the *T*. *cruzi* satellite tandem repeat assay performed on the ddPCR platform.

Operative characteristic	%	95% confidence interval	
Sensitivity	100.0	100.0	100.0
Specificity	100.0	100.0	100.0
Positive predictive value	100.0	100.0	100.0
Negative predictive value	100.0	100.0	100.0
Prevalence	55.2	48.2	62.2

The operative characteristics were determined including qPCR as a reference test [14]

The age of the patients was distributed normally (p = 0.1039), while the load (copies per μL) found by ddPCR was not distributed normally (p = 0.0000). Spearman correlation coefficient was determined and there was no correlation between the variables evaluated (age vs. parasitic load; Spearman's rho = 0.0012, p = 0.9900), and no correlation was found between the variables age and copies/μL quantified by qPCR (Spearman's rho = 0.0727, p = 0.4586). Finally, when evaluating the differences in median parasitic loads calculated by ddPCR between the two clinical phases, no statistically significant difference was found (p = 0.8399). Likewise, no statistically significant differences were found for the median parasitic loads calculated by qPCR between the phases (p = 0.9546). When evaluating the association by state with chronic infection cases, no statistically significant differences were found either by ddPCR or by qPCR (p = 0.5643 and p = 0.0567, respectively).

**Fig 5:**
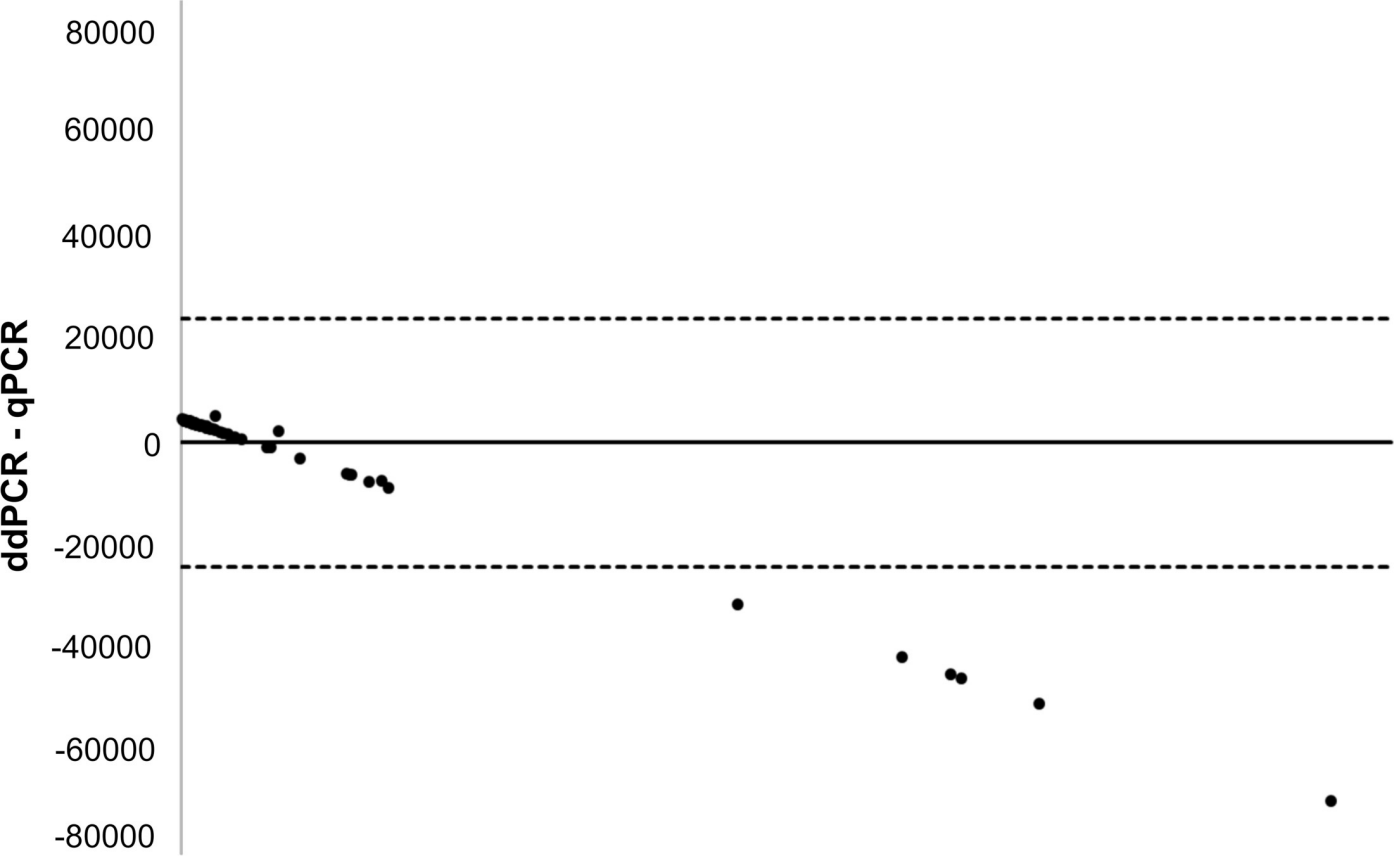
Bland-Altman plot showing the associations of parasitic load between qPCR and ddPCR.

A Bland–Altman graph was generated using Microsoft Excel 2016 software. No differences were observed in quantitation between the techniques when the parasitic loads were low. However, as the parasitic load increased, differences in the quantification between the two techniques began to become evident since ddPCR underestimates the actual parasitic load (Fig 5).

## Discussion

Chagas disease still presents a serious challenge to public health in Latin America and has increased in profile due to the immigration of infected individuals into non-endemic countries [35]. One of the challenges in controlling the disease is its diagnosis due to the stark contrast between the clinical signs of disease and the parasitic load between the acute and chronic phases of the disease [5]. Parasitological, serological methods and most recently molecular methods have been deployed to circumvent these challenges [10, 15, 36]. Due to the theoretical advantages of ddPCR outlined in the introduction, it is an attractive option for the detection and quantitation of low copy targets.

To optimize the performance of the assay on the ddPCR platform, we extended the number of cycles to 50 and increased the duration of both the dissociation and annealing/extension stages of thermocycling. This was successful in that it improved separation between positive and negative droplets, and vastly improved the ease of setting appropriate thresholds in this assay. One risk associated with increased cycle number is non-specific amplification, resulting in a loss in specificity. In this case the increase from 40 to 50 cycles had no effect on analytical specificity (as evidenced by consistent zero readings during repeat testing of the no-template control samples) and the excellent clinical specificity of the assay. Cycle number has also been increased to optimize assays in other contexts, such as the detection of genetically modified organisms [37], the detection of mutations in AH1N1-infected individuals [38] and the detection of foodborne pathogens in soft cheese [39]. One limitation of increasing the cycle number is the concomitant increase in the sample testing time; this may be problematic in high-throughput settings [31, 40].

We found the range of accurate quantitation of this assay to be between 10^0^−10^3^ parasites/mL, equivalent to approximately 5–5000 copies/μL. Typically, this would be considered to be within acceptable limits of performance for research purposes. The limit of detection of ddPCR was acceptable, and assay quantitation correlated well with parasitological findings (Pearson correlation coefficient: 0.987; efficiency: 98%; Fig 2). However, compared with qPCR, we could not demonstrate the theoretical improvement in precision at low loads that we expected. The CV was not significantly different between ddPCR and qPCR at any concentration tested [14]. However, it was interesting to observe that the within-plate CV of qPCR was higher at 1 parasite/mL than at 10 parasites/mL. This might be explained because at low concentration molecular tests tend to be unstable. This has been already reported for qPCR [14].

When comparing the limit of detection with that of qPCR, the limit of detection reported for this assay in the qPCR format was 0.46 parasites/mL [6], whereas in the ddPCR format, only 7/10 repeats were successfully detected at that level. This has been reported for other pathogens, where qPCR has been shown to be more sensitive, e.g. in Cytomegalovirus and *Xanthomonas citri* subsp. *citri* [22, 41]. There are a number of potential reasons for this, many of which are outlined by Hindson *et al*. [19]. The assay dynamics could be more suited to the qPCR well format. Also, the TcSTR is a variable region, as recently demonstrated, and its lack of stability could affect the sensitivity of a high-resolution test such as ddPCR [42]. One interesting finding was the difference between the dynamic range when testing epimastigotes versus trypomastigotes. To date, no studies have conducted standard curves with the human-infective stage of *T*. *cruzi*. Our results showed that the standard curve with trypomastigotes showed one order of magnitude more DNA than epimastigotes (Fig 3). This could be explained by the cell size of trypomastigotes as they may have double or triple the cell surface of epimastigotes and subsequently have more DNA content [43]. Also, it might be related to the genomic architecture of *T*. *cruzi* as it was recently shown that: i) the DNA transcripts change drastically between cell stages [44, 45], and ii) trypomastigotes present more telomeres and DNA content than epimastigotes using next generation sequencing [46, 47]. However, these assumptions need to be validated by conducting more experiments including the testing of metacyclic trypomastigotes from other DTUs. We are aware that one limitation of our study is that a strict analytical validation was not conducted as stated in international guidelines for clinical laboratories. However, this has not been done even for the qPCR or PCR platforms. We used the same pipeline of validation established for ddPCR platforms already published and it must be kept in mind that PCR platforms are not currently considered clinical diagnostic tools for Chagas disease.

When evaluating the diagnostic performance of this assay, the concordance between qPCR and ddPCR was found to be excellent (kappa = 1). With perfect agreement between the positives and negatives for each of the tests, the operational characteristics showed 100% in all cases (Table 2). The Bland–Altman plot also showed a good degree of association between the parasitic loads for both platforms (Fig 5). Although at high concentration, the ddPCR platform became saturated and qPCR showed a better performance, explaining the outliers from the graph. In general, this shows that in this sample set, despite not offering a technical performance benefit over qPCR, the use of this assay on the ddPCR platform may be as suitable as qPCR for the detection of *T*. *cruzi*. However, one study reported median parasitemia values in chronic Chagas disease patients from Argentina and Colombia to be 1.93 and 2.31 parasite equivalents/mL, respectively. Based on our present evaluation, the ddPCR assay should be able to reliably detect samples with these median loads but may fail on occasion with lower median parasitemia levels. More concerningly, the median load of parasitemia for Brazilian patients was 0.1 parasite equivalents/mL, which is below the reproducible limit of detection for the ddPCR assay [29]. A recent study of patients in the chronic phase showed a sensitivity of 64.2% and specificity of 97.1% for qPCR [6, 48], suggesting it too will not reliably detect all infections in chronic patients, resulting in a loss of sensitivity. Finally, the cost of ddPCR is known to be approximately double the price of qPCR, therefore without a significant performance improvement, it is unlikely that this constitutes a realistic public health surveillance tool. However, the primary utility of the ddPCR platform on which to run this assay is likely to be as a research tool.

In our study, the ddPCR assay was not efficient at high concentrations of *T*. *cruzi* (Fig 1). This impedes its utility in acute Chagas disease patients, where parasitemia levels tend to be high and would suggest that DNA samples would need to be diluted prior to application on a ddPCR platform. A possible solution might be exploring other molecular targets across the *T*. *cruzi* genome that may have a lower number of copies per cell; however, this could affect the sensitivity. Previously, some authors suggested the use of 18S, 24S, cytochrome oxidase II (COII) or the spliced leader of miniexon gene for the molecular diagnosis of *T*. *cruzi*. However, in an international consensus, these markers did not show satisfactory results in terms of sensitivity [13]. New experiments would need to be performed with these molecular targets under a ddPCR platform to determine their potential use. Additionally, in a recent analytical validation report for *T*. *cruzi*, the kinetoplast (kDNA) showed interesting results in terms of sensitivity that were improved for TcSTR [14]. We initially tested the kDNA reported by Ramírez *et al*. 2015 [14] and found oversaturation of the droplets preventing adequate discrimination of positive and negative droplets as with TcSTR. This might reflect the molecular nature of kDNA (discoid DNA with minicircles and maxicircles). Therefore, we did not pursue this marker. In Colombia, *Trypanosoma rangeli* is a frequent parasite and kDNA presents strong cross-reactivity with this protozoan [49]. Therefore, we concluded that TcSTR was the most suitable approach to initiate the analytical validation of a ddPCR platform. However, maybe an optimized primer or probe design could improve the analytical characteristics of ddPCR and additional experiments are needed to identify appropriate molecular candidates.

In several studies, ddPCR performed much better than qPCR for the detection of pathogen DNA (for example, *C*. *trachomatis*, *Salmonella*, *E*. *coli*, *C*. *jejuni*, cytomegalovirus and HIV) [20–24]. When applied to the detection of *Plasmodium* species, this tool was able to quantify low parasitemia in subpatent infections, as well as determine *Plasmodium* species in cases of low parasitemia [50]. In the case of *Cryptosporidium*, the precision of ddPCR was consistently higher compared with qPCR but decreased as the DNA concentration decreased [27]. We did not identify the same improvement in the detection of *T*. *cruzi* in blood samples after importing the assay to the ddPCR platform. However, this corresponds a pioneer study towards the establishment of an efficient protocol to employ ddPCR as a test to detect *T*. *cruzi* DNA in blood samples. As mentioned before, novel targets and better primer design might potentially improve the performance of the assay.

There are a number of limitations to our study. The samples used for the evaluation of clinical performance did not incorporate samples from other clinical settings outside of Colombia. This is important as the median parasitic load is higher in Colombian patients, and therefore the sensitivity may be reduced in patients from other regions. Also, the clinical stages, age and patient characteristics could impact on the results of ddPCR. In our case, we observed that *T*. *cruzi* DNA could be detected in acute and chronic patients by ddPCR with no statistical significance on the parasitic load in terms of age or clinical stage. However, verification is required in a novel group of patients including, for example, children and congenital cases. Additionally, the variation in TcSTR copy number between DTUs and even within DTUs may cause the results to be variable in clinical settings; a fixed copy number target would be preferable for the purposes of quantitation.

In conclusion, we report the first evaluation of the suitability of a ddPCR platform to detect *T*. *cruzi* DNA in blood samples. The analytical and diagnostic performance of the ddPCR assay evaluated here does not support its routine usage over qPCR. However, it shows several advantages such as quantitation of DNA without the need for calibration curves thereby allowing for exact and reference values to be obtained per sample (higher-order reference measurement method) as has been suggested for various viral diseases. A range of samples from a larger geographical area should be tested and a future international consensus should be considered as was conducted for qPCR to comprehensively determine the potential use of ddPCR to detect and quantify *T*. *cruzi* in clinical samples.
